# Green synthesis of chitosan nanoparticles using tea extract and its antimicrobial activity against economically important phytopathogens of rice

**DOI:** 10.1038/s41598-024-58066-y

**Published:** 2024-03-28

**Authors:** M. Sathiyabama, R. V. Boomija, S. Muthukumar, M. Gandhi, S. Salma, T. Kokila Prinsha, B. Rengasamy

**Affiliations:** Department of Botany, School of Life Sciences, Bharathidasan University, Tiruchirappalli, Tamil Nadu 620024 India

**Keywords:** Antimicrobial, Chitosan nanoparticles, Tea polysaccharides, Rice, *Pyricularia grisea*, *Xanthomonas oryzae*, Microbiology, Plant sciences

## Abstract

The aim of the present work is to biosynthesize Chitosan nanoparticles (CTNp) using tea (*Camellia sinensis)* extract, with potent antimicrobial properties towards phytopathogens of rice. Preliminary chemical analysis of the extract showed that they contain carbohydrate as major compound and uronic acid indicating the nature of acidic polysaccharide. The structure of the isolated polysaccharide was analyzed through FTIR and ^1^H NMR. The CTNp was prepared by the addition of isolated tea polysaccharides to chitosan solution. The structure and size of the CTNp was determined through FTIR and DLS analyses. The surface morphology and size of the CTNp was analysed by SEM and HRTEM. The crystalinity nature of the synthesized nanoparticle was identified by XRD analysis. The CTNp exhibited the antimicrobial properties against the most devastating pathogens of rice viz., *Pyricularia grisea, Xanthomonas oryzae* under in vitro condition. CTNp also suppressed the blast and blight disease of rice under the detached leaf assay. These results suggest that the biosynthesized CTNp can be used to control the most devastating pathogens of rice.

## Introduction

Global demand for food is predicted to increase by 70% in the next few decades which alarms the importance of sustainable agricultural production^1^. Rice (*Oryza sativa* L.) is one of the principal cereal crop which represents a staple food worldwide. Rice cultivation is adapted to a wide range of agro-climatic zones, hence FAO (Food and Agricultural Organization) regarded it as a strategic crop for food security^2,3^. However, rice production is constrained due to various diseases such as blast, leaf blight, sheath blight, sheath rot caused by phytopathogens^4^. Among them, *Pyricularia grisea* and *Xanthomonas oryzae* are the major destructive pathogens of rice, which cause blast, leaf blight (BLB) disease respectively and leads to severe yield losses^5,6^. They are widely prevalent among various rice varieties worldwide and both the pathogens infect the rice crop at all stages of growth, thus affecting the nutrient quality and quantity in terms of yield^5^. To control phytopathogens and to boost-up production, farmers use different types of chemical fertilizers and fungicides in paddy field^7^. The emerging plant pathogen resistance towards commonly used fungicides as well as potential threats of these agrochemicals in the ecosystem warrants effective control of plant pathogens through ecofriendly approach. Therefore, novel approaches that are ecofriendly and sustainable are necessary to control these phytopathogens.

Nanotechnology is one of the key technologies that promise to advance traditional agricultural practices for sustainable plant disease management^8–11^. The application of nanotechnology in agriculture for increased crop production was encouraged by FAO^12–14^. Nanomaterials have the potential to minimize the chemical input and protect crops from devastating phytopathogens^15–18^. Nanoparticles have been integrated into plant disease management approach as bactericides/fungicides to enhance plant and soil health^19–21^. Nanoparticles prepared from biopolymers have received much attention due to their biodegradability, biocompatibility and non-toxicity^22–25^. Chitosan, a cationic biopolymer obtained from crustaceans and its nanoparticles are very well documented for their broad spectrum antimicrobial properties towards phytopathogens^26–29^ and they also induce defense in various crops^30–32^. Chitosan nanoparticles are generally prepared chemically (besides physical method) by ionotrophic gelation using sodium tripolyphosphate (TPP). Though TPP is considered as safe it results in nanoparticles with variation in size, stability and biological property which affects its wide application in agriculture^27,33^. To minimize the size variation and to increase the stability, it is of necessary to identify an alternate green approach to prepare chitosan nanoparticles. Green synthesis offers advantage over traditional chemical and physical methods as it is environmentally acceptable and economically feasible method.

Tea (*Camellia sinensis* L.) and its extracts have been used as medicinal and dietary supplements to induce immunity, in many countries. Tea extracts are rich in bioactive compounds, such as polyphenols, anionic polysaccharides etc.^34–37^. In recent years, tea polysaccharides (TPS) received more attention due to their various biological functions^38^. This study reports the phytofabrication of chitosan nanoparticles through green approach using tea leaf extract (tea polysaccharides) as an alternative ecofriendly procedure. The green synthesized chitosan nanoparticles were characterized and evaluated for its antimicrobial property against phytopathogens of rice under in vitro condition.

## Results and discussion

### Characterization of tea polysaccharides

**Figure 1:**
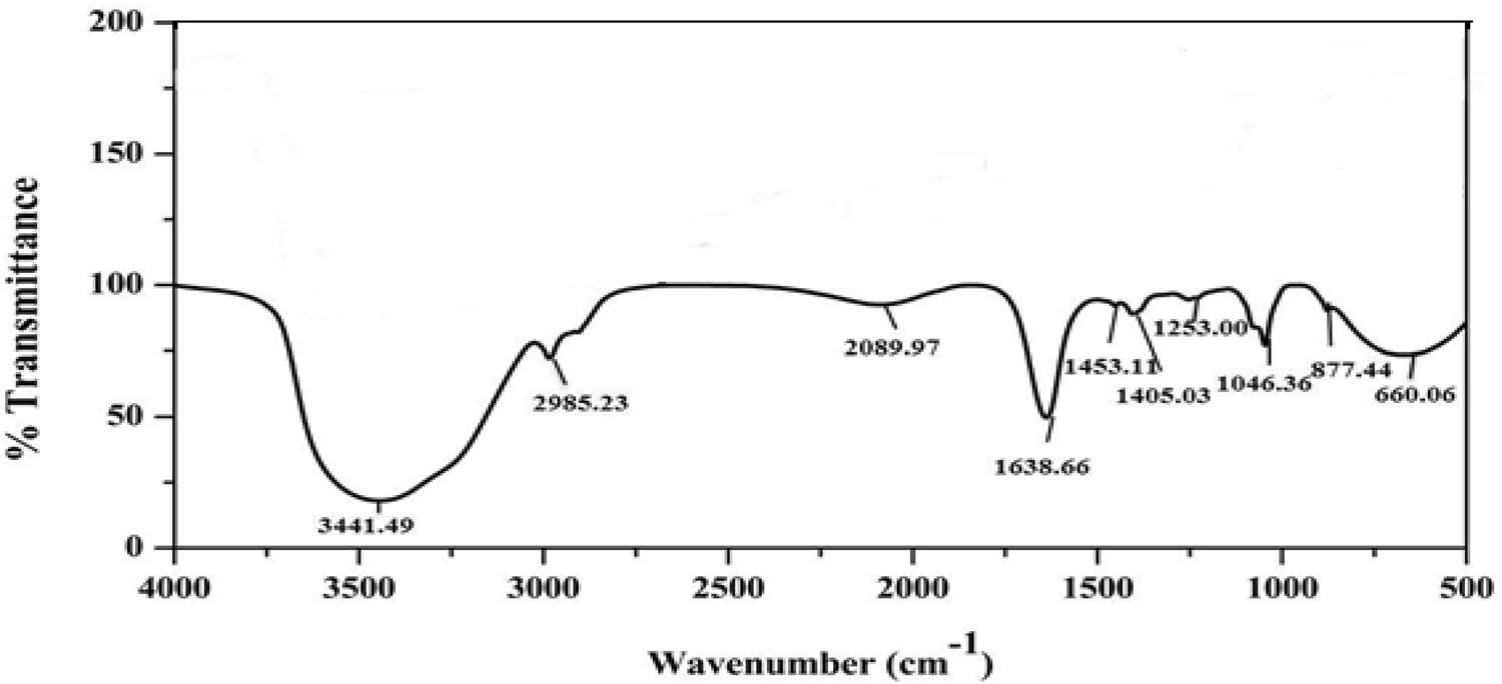
FTIR spectrum of Tea polysaccharide.

Tea polysaccharides were isolated from tea leaves using standard procedure and purified through DEAE-cellulose chromatography. The carbohydrate and protein content present in TPS isolated from leaves of *C. sinensis* was 89.4 ± 0.36% and 1.8 ± 0.3% of the total dry weight respectively. The uronic acid content was found to be 9 ± 0.2% indicating the nature of acidic polysaccharide. Preliminary TLC analysis indicates the presence of galactose, mannose, glucose, arabinose, xylose, and rhamnose in the polysaccharide (TPS) which is in accord with previous report^39^. FTIR spectrum indicates the presence of characteristic absorption peaks of polysaccharides (Fig. 1). A strong and broad absorption peak around 3441 cm^−1^, 2985 cm^−1^ due to O–H and C–H stretching vibrations respectively (Fig. 1) were observed. The absorption peak at 1638 cm^-1^ indicates C=O stretch and the presence of a carbonyl group of acidic polysaccharide^40^. The peak at 1453 cm^−1^ and 1405 cm^−1^ were attributed to C–H stretch of polysaccharides. The absorption peak at 1253 cm^−1^ due to C–O stretch indicates the existence of uronic acid characteristics of TPS. The glycosidic linkage stretch was observed at 1046 cm^−1^. The absorption peak at 877 cm^−1^ indicates α-glycosidic linkage in polysaccharide chains^37^.

**Figure 2:**
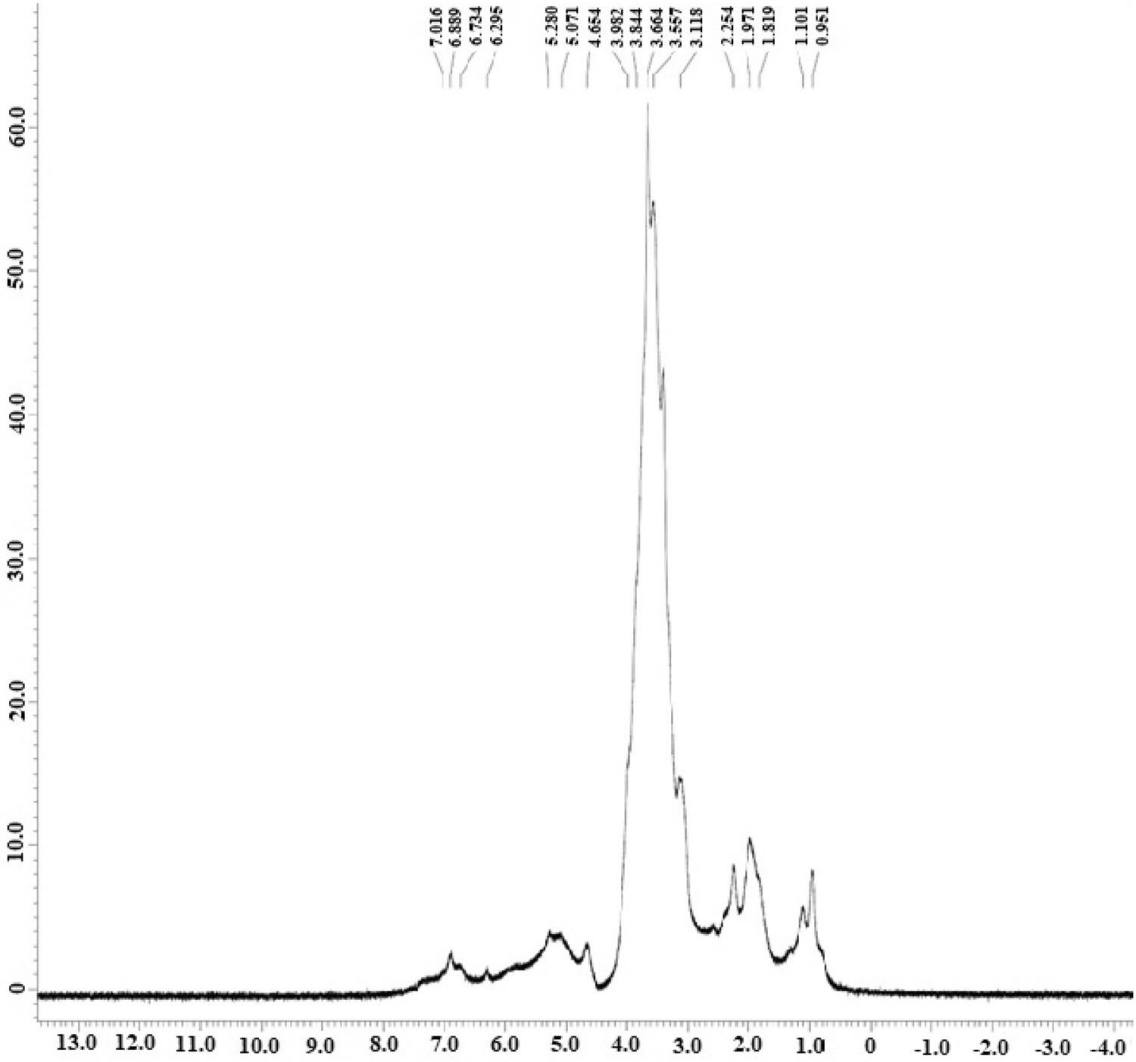
^1^H NMR spectrum of isolated TPS.

To confirm the presence of polysaccharides in the tea extract, ^i^H NMR was done. The ^1^H NMR analysis showed a set of wide and complex signals present between δH 3.1–5.2 ppm (Fig. 2) was characteristic of the typical polysaccharide signal^41^. Signals between 3.9–4.6 and 5.0–5.2 ppm confirmed the existence of both α and β configuration of saccharide residues^40,42^ which supports the result obtained from FTIR. These data suggest that the isolated product from tea leaf extract mainly composed of polysaccharides (TPS).

### Biosynthesis of chitosan nanoparticles

Of the various concentrations tried, TPS prepared at 2.5 mg/ml displayed the best result in forming the opalescent solution. The opalescent appearance observed after the addition of 27–30 ml of TPS to 100 ml of chitosan solution at the given temperature and pH. The TPS acts as a precursor/reducing agent and aided the formation of nanoparticles. It might be possible that anionic tea polysaccharide attaches to cationic NH_2_ groups of chitosan and convert them to chitosan nanoparticles. This bioreduction method involves single-step process and is eco-friendly, non-toxic. The formation of nanoparticles was primarily analyzed through UV–visible spectrophotometer which showed a single peak at 373 nm indicating the biofabrication of chitosan tea nanoparticles (CTNp). Green fabrication of chitosan nanoparticles using *Lavendula angustifolia* with antibiofilm activity has been reported^43^.

### Characterization of biosynthesized chitosan nanoparticles

#### FTIR analysis

**Figure 3:**
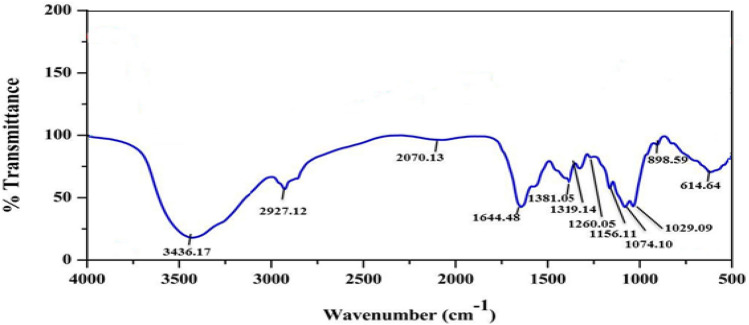
FTIR spectrum of biosynthesized CTNp.

FTIR analysis (Fig. 3) showed the presence of peak at 3436 cm^−1^ due to overlap of the OH and NH stretching. The typical peak at 2927 cm^−1^ due to C–H stretch indicates the structure of chitosan. The peak at 1644 cm^−1^ showed the presence of a carbonyl bond of amide group. The peak at 1319 cm^−1^ (N=O stretch), 1156 cm^−1^ indicates the binding of chitosan to TPS (Fig. 3). The presence of uronic acids was observed at 1260 cm^−1^. The glycosidic linkage was observed at 1029 cm^−1^ (C–O stretch). FTIR analysis indicates the structural stability of chitosan during the bioconversion of chitosan nanoparticles.

#### Dynamic light scattering analysis

**Figure 4:**
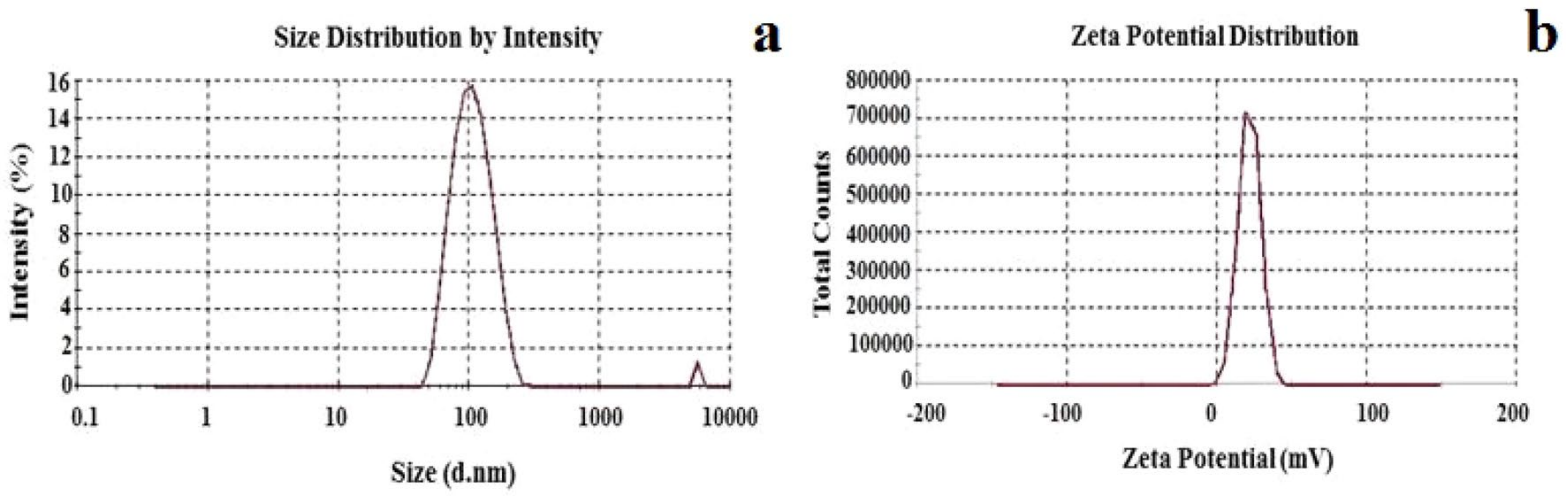
Dynamic Light Scattersing (**a**), Zeta Potential (**b**) of CTNp.

The diameter of the nanoparticle as measured by zetasizer was 118.7 nm with PDI value of 0.36 (Fig. 4a). This indicates the monodisperse nature of CTNp in aqueous solution which is vital for the biological activity^44^. The addition of acidic polysaccharides to chitosan solution aids the formation of chitosan nanoparticles, which might be due to simple electrostatic interaction. Surface charge identified through zeta potential analysis is an important parameter to evaluate the nanoparticle stability in suspension. For a physically stable nanoparticle suspension, a minimum zeta potential of ± 30 mV is required^45,46^. A high positive or negative zeta potential resists aggregation of nanoparticles in suspension^47^ due to the repulsion and confers stability. The zeta potential of the biosynthesized CTNp was observed to be + 33.1 mV (Fig. 4b). Higher positive ZP (+ 33.1 mV) of biosynthesized chitosan nanoparticles could exhibit substantial electrostatic repulsion between nanoparticles and thus prevents aggregation in aqueous phase. These results suggest that phytofabrication using tea extract yields monodispersed highly stable CTNp as compared to other methods^27,48^.

#### Electron microscopic and XRD analyses

**Figure 5:**
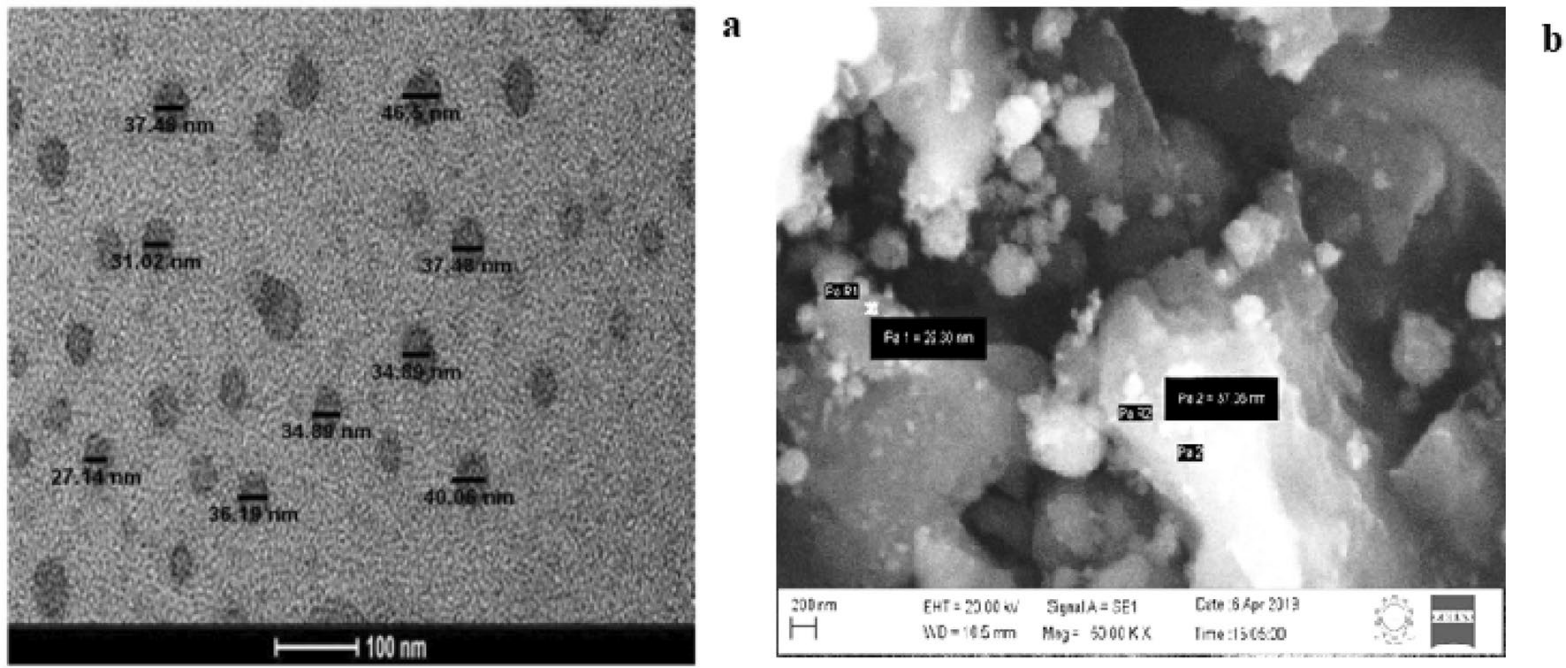
HRTEM image (**a**) and SEM image (**b**) of CTNp.

**Figure 6:**
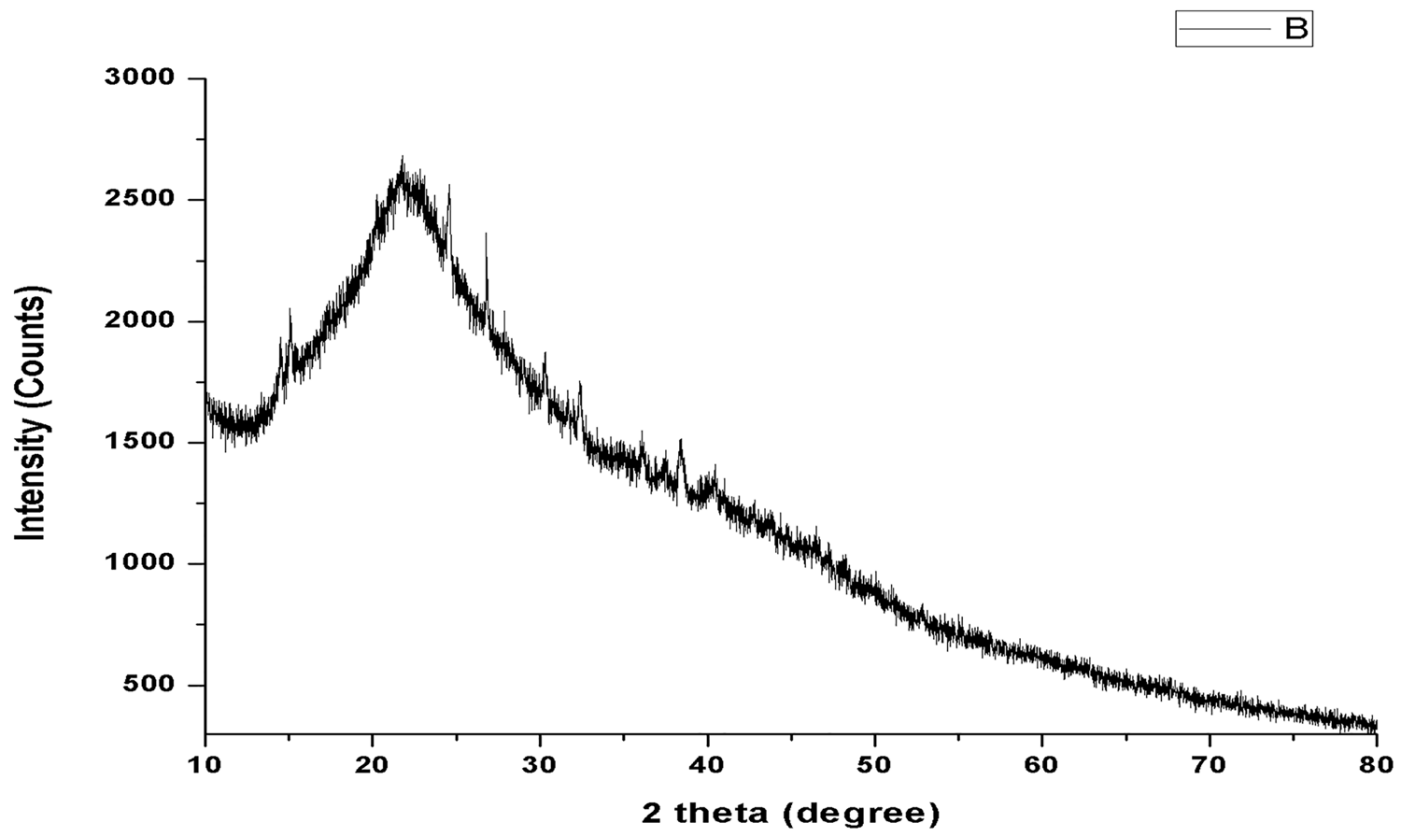
XRD pattern of CTNp.

HRTEM analysis showed well dispersed, spherical shaped nanoparticles with a size range between 25 and 40 nm (Fig. 5a). SEM micrograph revealed that the surface of nanoparticles was observed to be smooth and spherical (Fig. 5b). The size difference between TEM and DLS analyses may be due to different measurement conditions viz., dry and hydrodynamic state respectively involved in these techniques. The XRD analysis showed no peaks in the diffractogram (Fig. 6) implying the amorphous nature of nanoparticles, which augment the sorption properties of the materials.

#### Evaluation of antimicrobial activity of biosynthesized CTNp

**Figure 7:**
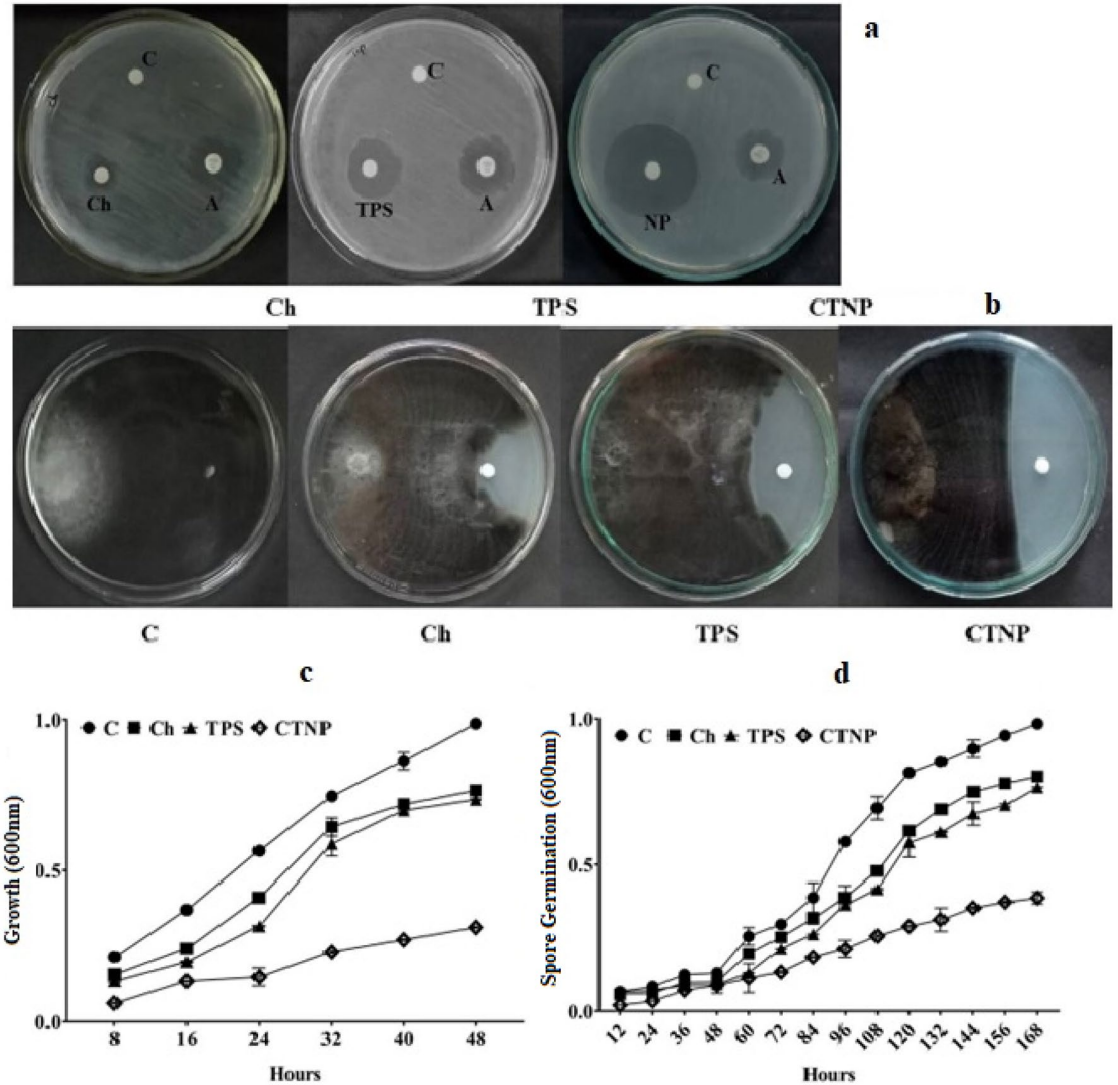
Antimicrobial activity of CTNp against *X. oryzae* (**a**, **c**) and *P. grisea* (**b**, **d**). C-Control, Ch-Chitosan, TPS-Tea polysaccharide, CTNp-Chitosan tea nanoparticle, A-Standard Antibiotic.

Biosynthesized CTNp and its individual bulk products (chitosan, TPS) used for synthesis of nanoparticles were evaluated for its antimicrobial potential against *X. oryzae* and *P. grisea,* the most devastating rice pathogens. Biosynthesized CTNp showed significant difference in growth inhibition of *X. oryzae* when compared to control, chitosan and TPS (Fig. 7a,c) under in vitro condition. Substantial inhibition (more than 90%) of hyphal growth and spore germination of *P. grisea* after exposure to biosynthesized CTNp (Fig. 7b,d) was observed. The bulk chitosan and TPS were found to be less effective in inhibiting the hyphal growth and spore germination of *P. grisea* under the same condition. These results indicated that biosynthesized CTNp was more effective in controlling both *P. grisea* and *X. oryzae* under in vitro condition. This may be due to its size/surface charge which makes CTNp as highly permeable towards the biological membrane. It is possible that the small size of CTNp makes easy entry through the fungal cell wall and binds to their DNA, proteins and inhibits the synthesis of mRNA^26^. The high positive surface charge of CTNp might interact with negatively charged biological membrane/cell wall of phytopathogens tested and alter the membrane permeability their by inhibits/control their growth. This was in accord with previous reports^29,49–51^.

#### In vivo leaf bioassay

**Figure 8:**
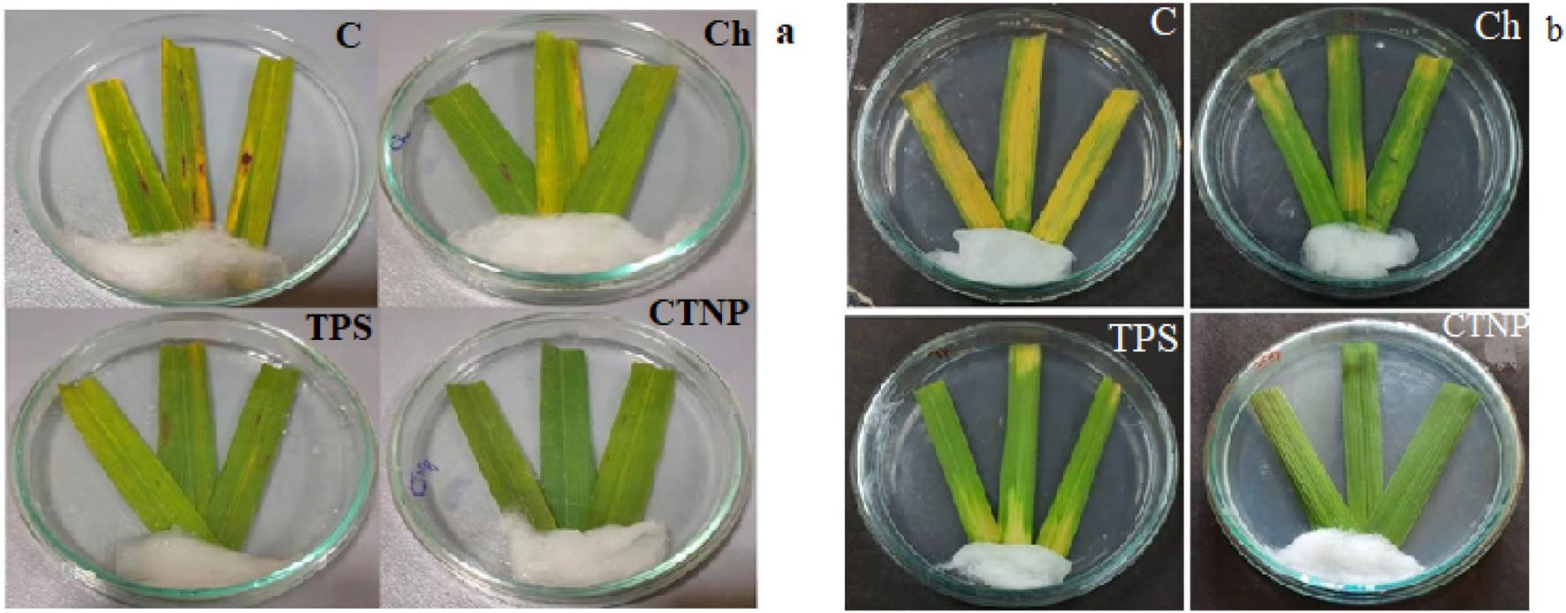
Blast (**a**) and Leaf Blight (**b**) disease suppression on detached leaf of *O. sativa*. C-Control, Ch-Chitosan, TPS-Tea polysaccharide, CTNp-Chitosan tea nanoparticle.

**Figure 9:**
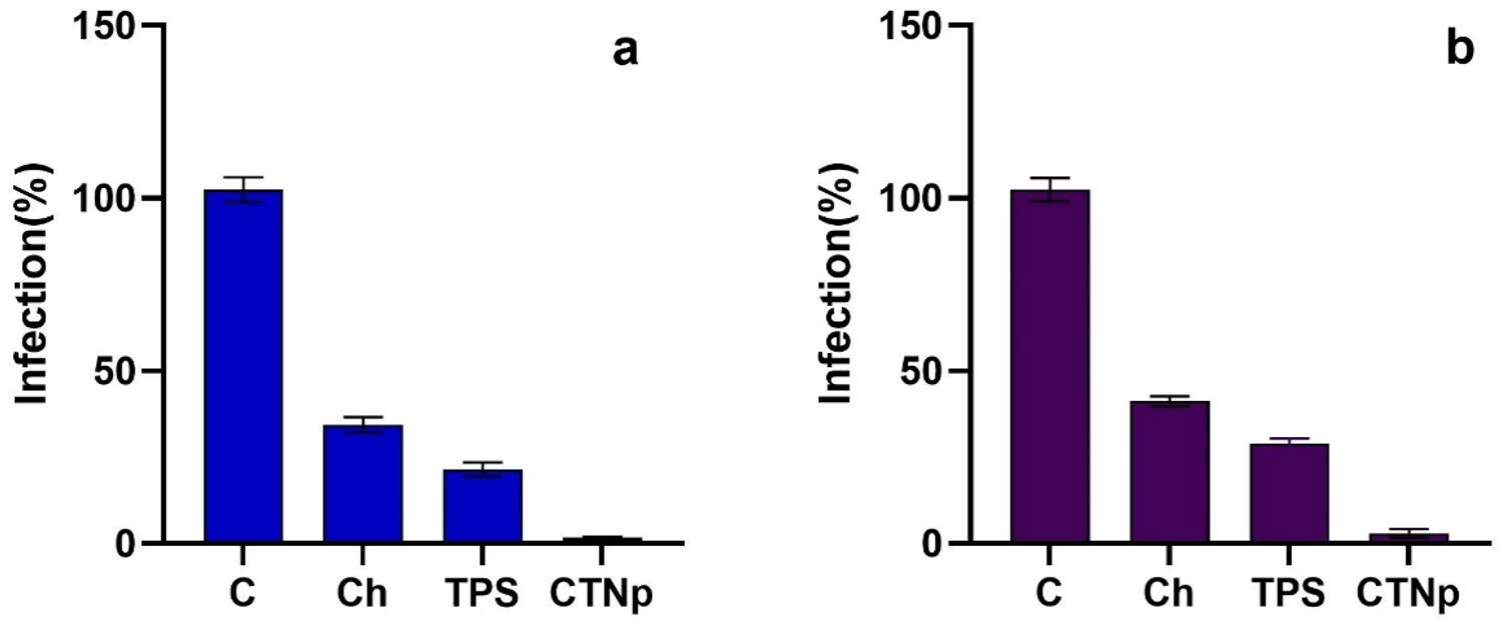
% infection of Blast (**a**) and Blight (**b**) disease on detached rice leaf. C-Control, Ch-Chitosan, TPS-Tea polysaccharide, CTNp-Chitosan tea nanoparticle.

The detached leaf method provides the in vivo atmosphere to the interacting phytopathogens and therefore exhibit similar results as of whole plants^48^. In chitosan, TPS treated leaves, the blast disease appeared after 4 days of infection whereas in control the symptom appeared after 2 days and progress further. Nearly 50% blast disease suppression was observed in chitosan and TPS treated leaves when compared to untreated infected control. In CTNp treated leaves, complete suppression of blast disease was observed in the test period (Figs. 8a, 9a). Likewise, the disease suppression was also observed for BLB disease in CTNp treated leaves when compared to its bulk counterparts (Figs. 8b, 9b). These results substantiate the biocidal property of biosynthesized CTNp. To our knowledge, this is the first report on plant disease suppression activity of TPS and CTNp. Inhibition of phytopathogenic fungus, *Botrytis cinerea* by green synthesized chitosan nanoparticles were reported recently^52^. CTNp can enter the plant system easily due to its small size through stomata, wounds, trichomes etc.^53,54^. Stomata serve as an efficient route for nanoparticle uptake^47,49^. Rice being a monocot has stomata on both sides^55^ which might have enhanced the rapid uptake of chitosan nanoparticles. Once it enters inside it might activate the defense response, their by control the growth of invading phytopathogens besides its biocidal property. In addition, the higher number of surface functional groups present in CTNp aids easy interaction with plant cells. The outcome of this work suggests that CTNp can be green synthesized using tea extract and can be used in the agricultural sector for disease management. Further work is necessary to evaluate the biosynthesized CTNp in protecting rice crops under field conditions.

## Conclusion

This study reports the biosynthesis of chitosan nanoparticles using the extract of *C. sinensis*. The tea extract was partially purified through DEAE cellulose column chromatography. Preliminary chemical analysis indicates it as acidic polysaccharide. The structure was analyzed by FTIR and ^1^H NMR. The biosynthesized chitosan nanoparticles were characterized through FTIR, HRTEM, SEM, DLS and XRD analyses. The zeta potential of the biosynthesized CTNp was observed to be + 33.1 mV, indicating its stability in aqueous solution. CTNp showed inhibitory activity against the most devastating rice pathogens viz., *P. grisea* and *X. oryzae* under in vitro condition. Detached leaf method showed suppression of the blast, blight diseases of rice and suggests that CTNp can be used to control rice phytopathogens. However, this requires field evaluation to establish.

## Materials and methods

### Preparation and characterization of tea extracts

Tea polysaccharide was extracted following the procedure of^34^. Tea (*Camellia sinensis* L.) was purchased (black tea; includes both leaves and buds) from the local market, extracted (1 g/50 ml) with water at 85 °C for 3 h and cooled to room temperature. The extract was concentrated to 5 ml using rotary vapor with continuous rotation in a water bath set at 37 °C and it was further extracted twice with equal volume of chloroform: n-butanol (4:1, v/v) to remove the contaminating proteins. To the aqueous phase, two volumes of ice cold ethanol (95%) was added, kept at 4 °C overnight and centrifuged at 12,000 G for 15 min (Eppendorf 5415R, Germany) to precipitate the polysaccharides. The precipitate was washed twice with ethanol, dissolved in sterile distilled water, dialyzed against distilled water for 24 h at 4 °C and freeze dried. The extract (0.5 g/ml) was then passed through DEAE-cellulose column chromatography (30 × 2.5 cm diameter; at room temperature) pre-equilibrated with sterile distilled water. The sample was sequentially (3 ml/fraction) eluted using distilled water with 0.1–0.5 M NaCl gradient solutions. Fractions were tested for the presence of carbohydrate by the Dubios method. Fractions showing peaks were pooled together dialyzed against distilled water (to remove the salt) at 4 °C for 24 h, freeze dried and used for characterization.

Total polysaccharide content was quantified by the phenol–sulphuric acid method following Dubios et al.^56^ using galactose as a standard. Protein content was analyzed by Bradford^57^ method using BSA fraction V as a standard. Uronic acid was determined based on Blumenkrantz and Asboe-Hansen^58^ method. The crude polysaccharide (1 mg) was hydrolyzed with 200 µl of 3 M trifluroacetic acid at 120 °C for 4 h. The hydrolyzates were centrifuged and separated (10 µl) on silica gel coated TLC (0.2 mm thickness) plates along with standards (Sigma Chem. Co., USA) using n-butanol: pyridine: water (50:28:32) as solvent system. The plates were air dried, sprayed with aniline phthalate reagent and Rf values were calculated. FTIR analysis was performed in Nicolet 560 FTIR spectrophotometer in a frequency range between 400 and 4000 cm^−1^ using the KBR pellet method. The ^1^H NMR spectra were recorded on a Bruker DRX-500 MHZ spectrophotometer using D_2_O as solvent system.

### Biosynthesis of chitosan nanoparticles (CTNp)

Chitosan (85% acetylation; molecular weight 27 kDa; 250 mg/100 ml) dissolved in 0.1% acetic acid and pH was adjusted to 4.2. The tea extracts (TPS) were prepared separately (0.5, 1.0. 1.5, 2.0, 2.5, 3.0 mg/ml) in sterile distilled water and added to chitosan solution drop by drop under stirring at room temperature till the appearance of opalescent solution. Further, it was allowed under magnetic stirring for 2–3 h and the colloidal solution was centrifuged at 15,000 G for 30 min. The precipitate was washed with 70% (v/v) ethanol and freeze dried.

### Physico-chemical characterization of biosynthesized chitosan tea nanoparticles

UV–visible spectra were recorded (200–400 nm) using Shimadzu UV–visible 1800 Spectrophotometer for preliminary confirmation of nanoparticle formation. FTIR was performed by KBr pellet method in the region of 400–4000 cm^−1^, to identify the functional groups in the nanoparticles using Nicolet 560 FTIR spectrophotometer. Particle size, surface charge and poly dispersity index (PDI) were analyzed by DLS using Zetasizer (Malvern, UK) at 25 °C in triplicate. The nanoparticle size and surface morphology were examined by HRTEM (JOEL model, 1200 Ex) and SEM (Zeiss, Evo 18, Germany) respectively. X-ray diffraction studies were performed in X-ray diffractometer (Rigaku Ultima III XRD) with a CuKα1 radiation to determine the structure of the sample. The X-ray source was operated at 40 kV and 40 mA. Diffraction intensity was measured in the reflection mode at a scanning rate of 2/min for 2θ = 10°–70°.

### Determination of antimicrobial activity

The biosynthesized chitosan nanoparticles were evaluated for its antimicrobial property against the most devastating rice pathogens viz., *Pyricularia grisea* and *Xanthomonas oryzae* under in vitro condition.

### Antifungal activity

The mycelial disc (7 mm diameter) was removed from the actively growing end of seven day old culture of *P. grisea*, placed upside down position in a petri plate containing potato dextrose agar (PDA) medium. To its opposite, a sterile filter disc (6 mm diameter) impregnated with (100 µg/100 µl) chitosan, TPS, CTNp was placed separately and incubated at 25 ± 2 °C for 10 days. For control, filter discs were added with sterile distilled water. Experiments were done in triplicate and repeated thrice. % of inhibition was calculated as follows:

$$ Inhibition\,\left( \% \right) \, = \frac{{Growth\,\left( {mm} \right)\,in\,control\,{-}\,Growth\,\left( {mm} \right)\,in\,treated}}{{Growth\,\left( {mm} \right)\,in\,control}} \times 100 $$

For spore assay, spore suspension (1 × 10^5^ spores/ml) of *P. grisea* was prepared in sterile distilled water from 10 days old cultures grown in potato dextrose agar medium. Czepak’s Dox broth (half strength) medium amended with (0.1%, w/v) chitosan, TPS, CTNp were prepared separately. To this, the spore suspension (5 ml/50 ml medium) was added and incubated in an orbital shaker at 27 °C with 120 rpm for 7 days. Optical readings were taken at 600 nm at every 12 h interval up to 7 days. The experiments were repeated thrice with triplicates.

### Antibacterial activity

Antibacterial activity (*X. oryzae*) of synthesized chitosan nanoparticle (CTNp) was done by disc diffusion method using Muller-Hinton agar (MHA) medium. Actively growing (over night grown) culture of *X. oryzae* (100 µl) was added in the centre of the petri plate and uniformly spread over the medium. Sterile filter disc (6 mm diameter) impregnated with (100 µg/100 µl) chitosan, TPS, CTNp was placed in the medium along with standard antibiotics. For control, sterile distilled water was added to the disc. The petri plates were incubated at 37 °C for 24 h. For each sample, five plates were used and repeated thrice. % of inhibition was calculated using the formula:

$$ Inhibition\,\left( \% \right)\, = \,\frac{{Radial\,growth\,\left( {cm} \right)\,in\,control\,{-}\,Radial\,growth\,\left( {cm} \right)\,in\,treated}}{{Radial\,growth\,\left( {cm} \right)\,in\,control}} \times 100 $$

For spectrophotometrical assay, nutrient broth (half strength) medium amended with (0.1%, w/v) chitosan, TPS, CTNp were prepared separately. To this, active *X. oryzae* culture (1 ml/10 ml medium) was added and incubated in an orbital shaker at 37 °C with 120 rpm for 48 h. Optical readings were taken at 600 nm at every 8 h interval up to 48 h. The experiments were repeated thrice with triplicates.

### In-planta leaf bioassay

To evaluate the effect of CTNp on blast and leaf blight disease suppression, detached leaf bioassay was done. Collection of plant material, complied with relevant institutional, national, and international guidelines and legislation. Rice plants were grown in pots under in vitro condition. Leaves from 30 d old rice plants were washed thoroughly in running tap water followed by sterile distilled water. The leaves were treated (both abaxial and adaxial surfaces) with 0.1% (w/v) chitosan, TPS, CTNp separately (1 ml/leaf) using a paint brush, placed in sterile petri plate and incubated at 25 ± 2 °C for 24 h. For control, water was used. After 24 h, the control and treated leaves were inoculated with (1 ml/leaf) *P. grisea* spore suspensions (1 × 10^5^ spores/ml) using a paint brush. The plates were maintained at 25 ± 2 °C with 90% humidity for 10–14 days to monitor the blast disease development.

Another set of experiments was performed using *X. oryzae*. *X. oryzae* was grown in nutrient broth over night and the culture was harvested (1 O.D at 600 nm). Treated and control leaves (as above) were infected with *X. oryzae* (1 ml/leaf) and the plates were incubated at 25 ± 2 °C. The leaf blight disease development was monitored for the next 14 days.

### Statistical analysis

All the data were subjected to one-way analysis of variance (ANOVA) to determine the significance of individual differences in *p* < 0.01 and 0.05 levels. All statistical analysis was conducted using SPSS 21 software support version 5.0.

## Data Availability

All data generated during this study are included in this article.
